# Anti-photoaging effects of flexible nanoliposomes encapsulated *Moringa oleifera Lam*. isothiocyanate in UVB-induced cell damage in HaCaT cells

**DOI:** 10.1080/10717544.2022.2039802

**Published:** 2022-03-11

**Authors:** Yijin Wang, Qianqian Ouyang, Xuefei Chang, Min Yang, Junpeng He, Yang Tian, Jun Sheng

**Affiliations:** a College of Food Science and Technology, Yunnan Agricultural University, Kunming, China; b Marine Biomedical Research Institution, Guangdong Medical University, Zhanjiang, PR China; c The Marine Biomedical Research Institute of Guangdong Zhanjiang, Zhanjiang, China; d Engineering Research Center of Development and Utilization of Food and Drug Homologous Resources, Ministry of Education, Yunnan Agricultural University, Kunming, China; e Yunnan Province Engineering Research Center of Functional Food of Homologous of Drug and Food, Yunnan Agricultural University, Kunming, China

**Keywords:** *Moringa oleifera* seed isothiocyanate, hyaluronic acid-ceramide, flexible nanoliposomes, anti-photoaging

## Abstract

Skin photoaging is premature skin aging damage that occurs after repeated exposure to ultraviolet (UV) radiation. Although isothiocyanates extracted from the moringa tree (*Moringa oleifera Lam.*) (MITC) exhibit excellent effects against skin photoaging, its application is restricted because of its characteristics, such as extremely low water solubility, bioavailability, and easy degradation. Currently, flexible nanoliposomes have gained increasing interest as a biocompatible polymer for applications such as transdermal drug delivery. We prepare amphiphilic hyaluronic acid (HA) conjugated with ceramide (CE) to modify nanoliposomes for MITC (HACE/MITC NPs) delivery. The HACE/MITC nanoparticles (NPs) are prepared and characterized for entrapment efficiency, particle size, polydispersity index, zeta potential, *in vitro* release, *in vivo* skin permeation, and *in vitro* protective effect of photoaging. The zeta potential of MITC NPs and HACE/MITC NPs is −24.46 mV and −24.93 mV, respectively. After modification of HACE, the entrapment efficient of MITC liposome increased from 62.54% to 70.67%, and the particle size decreased from 266.1 nm to 192.8 nm. *In vivo* skin permeation, permeated drug increased from 49.42 to 71.40%. Moreover, the results showed that the entrapment of MITC in nanoliposomes improves its stability, efficacy, and skin permeation. Further, HACE/MITC NPs are favorable for uptake by HaCaT cells without requiring changes in cell morphology, which significantly improves the activities of antioxidant enzymes, scavenges UVB-induced reactive oxygen species, protects skin from damage, and reduces MMP-1, MMP-3, and MMP-9 expression caused by radiation-induced photoaging. Our results strongly suggest that flexible nanoliposomes successfully improved the cell membrane permeation of MITC, and that anti-photoaging and HACE/MITC NPs can potentially be used as candidates for photoaging therapy.

## Introduction

Ultraviolet (UV) radiation is harmful to human skin and causes tanning, sunburn, immune suppression, cancer, and photoaging (Afnan et al., 2012). Skin photoaging is a common skin disease and its common clinical manifestations include skin elasticity loss, epidermal atrophy, deep wrinkles, and normal pigmentation, which can lead to cell necrosis, apoptosis, or cancer (Walton et al., 2005; Kim et al., 2011). Skin photoaging seriously affects not only the physical appearance and mental quality of life, but it also has an etiological association with the occurrence of skin cancer. Therefore, skin photoaging caused by UV and its related issues has gained considerable interest as a future research topic. Chronic exposure to UVB (280–315 nm) radiation results in the enhanced generation of reactive oxygen species (ROS) in the skin, which leads to oxidative stress and photodamage to macromolecules such as proteins and nucleic acids (Jo et al., 2012). Further, such chronic exposure can cause adverse changes in the extracellular matrix (ECM), which lead to skin photoaging because of the enhanced production of matrix metalloproteinases (MMPs) such as MMP-1, MMP-3, and MMP-9, which is possibly mediated by the augmented ROS production (Gęgotek et al., 2021).

Seeds from the moringa tree (*Moringa oleifera Lam.*) contain rich isothiocyanates (MITC) that emerged as highly stable analogues because of the additional sugar moiety present in the aglycone portion of the molecule. Isothiocyanates are organic compounds with a –N═C═S group in their structures (Waterman et al., 2014), To be specific, we used 4-[(α-l-rhamnosyloxy) benzyl] isothiocyanate to complete experiment. The compound is easy to decompose under light or normal temperature. Studies have indicated that MITC exhibit excellent antimicrobial, anti-inflammatory, antioxidant, and antitumor activities (Tumer et al., 2015). Our previous study showed that MITC had an excellent effect on skin photoaging; however, its application in anti-photoaging cosmetics remains limited because of its easy decomposition under light and heat. Further, MITC is a fat-soluble compound with poor water solubility, which leads to low bioavailability. In addition, the effect of the skin barrier hinders the efficient delivery of drugs, thereby making it difficult to predict the transdermal process. In previous studies, the nanodelivery systems of isothiocyanates have been concerned (Wang & Bao, 2021). But researchers have focused more on the development of oral isothiocyanate as an anticancer nanodelivery system. There is little research on the transdermal delivery system of isothiocyanate.

Nanoliposome materials are widely used because of their good biocompatibility and biodegradability (Brys et al., 2016). The solubility and bioavailability of drugs can be increased when drugs are loaded with nanoliposome materials, which makes it easier to capture drugs at the lesion site (Vahabi & Eatemadi, 2017). Compared with other natural polymers, nanoliposomes are widely used as nanocarriers to control mucosal adsorption and promote the absorption of targeted carriers for slow drug release because of characteristics of bioadhesion and pellet formation (Abumanhal-Masarweh et al., 2019). Further, the rapid uptake of drug-loaded nanoparticles (NPs) by skin cells is achieved through endocytosis. This allows the drug to be released after entering the cells, and the local effect is more significant. This study intends to wrap MITC in nanoliposomes to reduce the barrier effect of the skin cuticle on MITC and provide MITC skin adhesion and slow release properties to improve its cumulative infiltration. This magnifies and improves the anti-photoaging effect of MITC.

Hyaluronic acid (HA), which has excellent biocompatibility and biodegradability, can be applied to several methods of drug delivery. In addition, ceramide (CE) is a known cellular signaling molecule that is involved in the regulation of differentiation, proliferation, programmed cell death, and apoptosis (Chen et al., 2010; Choi et al., 2011). Thus, in this investigation, amphiphilic HA conjugated with CE was prepared to modify the nanoliposomes for MITC (HACE/MITC NPs) delivery. The protective properties of HACE/MITC NPs against UVB-induced photoaging have been ascertained in human immortalized keratinocytes (HaCaT) cells, which implies its potential application as photoaging therapy candidate.

## Materials and methods

### Materials

*Moringa oleifera Lam* seed isothiocyanate was prepared by the Development and Utilization of Food and Drug Homologous Resources Engineering Research Center of the Ministry of Education (Kunming, China). Soy lecithin (95%), DS-Y30, HA, and cholesterol and sodium cholate were purchased from A.V. T. (Shanghai) Pharmaceutical Co., Ltd. (Shanghai, China), Evonik Degussa China Co., Ltd. (Beijing, China), Shanghai Yuan Bio Technology Co., Ltd. (Shanghai, China), and Shanghai Macklin Biochemical Co., Ltd. (Shanghai, China). Further, chloromethylbenzoyl chloride, tetra-n-butylammonium hydroxide (TBA), acetonitrile, dichloromethane, trichloromethane, and methanol were obtained from Shanghai Macklin Biochemical Co., Ltd. (Shanghai, China).

The cell culture medium (DMEM) and heat-inactivated fetal bovine serum (FBS) were purchased from Gibco BRL Life Technologies, Inc. (Grand Island, NY). Penicillin–streptomycin liquid, trypsin–EDTA solution 0.25% (without phenol red) and 0.01 M PBS (powder, pH 7.2–7.4) were purchased from Beijing Solarbio Science & Technology Co., Ltd. (Beijing China). The antibodies were obtained from Proteintech Group Inc. (Chicago, IL). All other chemicals were of analytical grade; ultrapure water was used throughout the entire study.

### Preparation of isothiocyanate-loaded nanoparticles formulations

The HA and CE were grafted according to the method reported by Cho et al. (2011); the composition of the formulations is listed in Table 1. Blank, HAE-modified liposomes, MITC-loaded, and HAE-modified MITC-loaded liposomes were prepared by a thin-film hydration method. For the blank liposome, lecithin (460 mg), cholesterol (46 mg), and sodium cholate (44 mg) were dissolved in methanol and trichloromethane (1:1, v/v) (Table S1). The mixture was transferred to a round-bottom flask after the complete dissolution of the solution. The mixture of methanol and trichloromethane was removed by rotary evaporation (YRE-2000E rotary evaporator, YUHUA, Shanghai, China) under vacuum at 37 °C to form a phospholipid film, diluted with pH 7.4 of PBS buffer (10 mL), and added to a thin film-coated flask and hydrated at 25 °C for 12 h. The resulting preparation was sonicated with a sonicator (CR-100S, CHUNLIN, Shanghai, China) for 7.78 min (478 s). This method was used for all liposomes, with minor differences in the additions once the phospholipid film was obtained: For the HACE modified liposome, HACE (30 mg) diluted with a PBS buffer (pH 7.4, 10 mL) was added to a thin film-coated flask and hydrated at 25 °C for 12 h; for the MITC-loaded liposome, MITC (0.5 mM, 1 mL) diluted with PBS buffer (10 mL) was added to a thin film-coated flask and hydrated at 25 °C for 12 h; and for the HACE-modified MITC-loaded liposome, HACE (30 mg) and MITC (0.5 mM, 1 mL), diluted with PBS buffer (pH 7.4, 10 mL), were added to a thin film-coated flask and hydrated at 25 °C for 12 h. These liposomes were stored at 4 °C in the dark.

**Table 1.** Characterization of liposomes formulation.

Composition	Mean diameter (nm)	Polydispersity	Zeta potential (mV)	Encapsulation efficiency (%)
Black liposome	221.43 ± 14.06	0.36 ± 0.03	–23.76 ± 0.46	–
HACE liposome	198.07 ± 15.70	0.31 ± 0.06	–25.36 ± 0.76	–
MITC-loaded liposome	266.1 ± 34.62	0.34 ± 0.07	–24.46 ± 0.53	62.54 ± 5.54
HACE MITC-loaded liposome	192.80 ± 2.50	0.308 ± 0.01	–24.93 ± 0.56	70.67 ± 0.42

Values are presented as mean ± standard deviation (SD) (*N* = 3).

### Characterization of nanoparticles

Nuclear magnetic resonance hydrogen spectroscopy (^1^H NMR) and Fourier transform infrared spectroscopy (FTIR) were used to confirm whether HA was modified from CE IIIB. Nanoliposomes were characterized by particle size, polydispersity index, and zeta potential, and these characterizations were performed using a Malvern Zetasizer Nano ZS90 (Zetasizer Nano ZS90, Malvern Instruments, Worcestershire, UK). Finally, the morphologies of NPs, MITC/NPs, HACE/NPs, and HACE/MITC NPs were imaged using a transmission electron microscopy (TEM, JEOL JEM 2100F, Tokyo, Japan).

### Encapsulation efficiency

The encapsulation efficiency (EE%) of MITC was measured using a low-temperature ultracentrifugation method (Liu et al., 2017). The free MITC was separated from the NPs by ultracentrifugation. In brief, NPs were diluted three times, then centrifuged at 4 °C and 15,000 rpm for 20 min. Methanol was added into the supernatant to detect the concentration of free MITC (i.e. *C*_free_). Further, NPs were diluted three times and methanol was added to break the emulsion, the mixture was centrifuged at 4 °C and 15,000 rpm for 20 min. The concentration of supernatant was denoted by *C*_total_. The concentrations were measured using a UV spectrophotometer (Aoyi, Shanghai, China). The concentration of MITC was determined using a spectrophotometer at an absorption wavelength of 236 nm. The EE% is expressed as

$$\text{EE\%}=(1\;-\frac{C_{\mathrm{free}}}{C_{\mathrm{total}}})\times \;100\%$$

### *In vitro* isothiocyanate release from nanoparticles

The MITC release from MITC NPs, HACE/MITC NPs, and pured MITC were assessed *in vitro*. One milliliter of MITC NPs, HACE/MITC NPs, and pured MITC were placed in dialysis bags which were then placed in 40 mL PBS (pH 7.4/pH 5.5). The release pattern was monitored for 48 h at 25 °C with shaking at 100 rpm. The release medium (4 mL) was collected at predetermined intervals, the absorbance was measured, and the concentration was calculated. An equal amount of fresh PBS (4 mL) was added to the release medium. The cumulative drug release of MITC from the MITC/NPs, HACE/MITC NPs and pured MITC was calculated using:

$$\text{Cumulative release \%}=\frac{C_{\text{release medium}}\times V_{\text{release medium}}}{C_{\text{added in nanoparticles}}\times V_{\text{added in nanoparticles}}}\times 100\%$$

where *C*_release medium_ is the MITC concentration in the release medium; *C*_added in nanoparticles_ is the MITC concentration of NPs added in the dialysis bags; *V*_release medium_ is the sample volume of the release medium; *V*_added in nanoparticles_ is the volume of the NPs in the dialysis bags.

### Stability of MITC

The MITC sample (104 μg/mL) and liposome loaded with MITC were evaluated for stability by UV spectrophotometry at different time points (0, 0.5, 1, 6, 12, 24, 48, 72, 120, 168, and 240 h). The samples were stored at room temperature (25 °C) and kept away from light.

### *In vitro* skin permeation

Healthy male C57 mice (6 weeks old) were sacrificed via cervical dislocation, and subcutaneous mucosal fat was removed carefully after shaving off the back hair and stripping off the back skin. The experiment was performed in accordance with guidelines and the ethics approval of Experimental Animal Care and Use Committee of Guangdong Medical University (production certificate number: SCXK (Yue) 2020-0147). The skin was washed with physiological saline and dried before use. The skin was mounted on Franz’s diffusion vertical cells (Huanghai, Shanghai, China). The cuticle of the skin was squeezed upward, MITC NPs or HACE/MITC NPs (1 mL) were added to the donor medium, the receptor compartment was filled with 7 mL physiological saline to immerse the dermis of the skin completely (no bubbles), the magnetic stirring was set at 500 rpm, and the temperature of water was set to 37.0 ± 0.1 °C. Samples were collected at 1, 2, 4, 6, 8, 12, 16, and 24 h and 0.2 mL physiologic saline was added in the receptor compartment. Drug accumulation was quantified using a UV spectrophotometer (Aoyi, Shanghai, China). After the percutaneous absorption test, skin specimens were detected by grinding and centrifugation to determine the drug content.

### Cellular uptake study

Cellular uptake and distribution of the drug (MITC) were evaluated in HaCaT cells by confocal laser scanning microscopy (CLSM). For the CLSM study, cells were seeded in a 24-well plate at a density of 1 × 10^4^ cells/well and then cultured in 600 μL DMEM containing 10% FBS for 24 h at 37 °C. FITC was loaded onto MITC/NPs and HACE/MITC NPs as the fluorescent dye (Wang et al., 2020). After removing the cell culture media, MITC/NPs and HACE/MITC NPs, which correspond to 20 μg/mL drug, were added and incubated for 2 h. The cells were washed thrice with PBS (pH 7.4). Then, DAPI (4′,6-diamidino-2-phenylindole; Biyuntian Biotechnology Co., Ltd., Shanghai, China) was added to stain the nuclei for 20 min at 25 °C; the cells were washed three times with cold PBS, fixed with 4% formaldehyde for 15 min, followed by the removal of 4% formaldehyde and addition of cold PBS. The cellular uptake and distribution were visualized using an Olympus IXplore SpinSR (Olympus, Tokyo, Japan).

### *In vitro* cytotoxicity tests of nanoparticles

The *in vitro* cytotoxicity of MITC, MITC NPs, HACE NPs, and HACE/MITC NPs toward HaCaT cells was studied using the MTT assay (Ron-Doitch et al., 2016). HaCaT cells were seeded in a 96-well plate at a density of 1 × 10^4^ cells/well and cultured in 200 μL of DMEM containing 10% FBS for 24 h. The MITC, MITC NPs, HACE NPs, and HACE/MITC NPs were added and cultivated for 24 and 48 h, respectively. Subsequently, the NPs were removed. The medium was then replaced with fresh DMEM and MTT solution at a ratio of 10:1. After 4 h of incubation, the medium was removed, and DMSO (100 μL) was added. The mixture was shaken for 10 min, and the absorbance at 570 nm (OD570) was measured using a microplate reader. Fresh DMEM was used as a control. The relative cell viability was calculated using:

$$\text{Cell viability }(\%)=\frac{\mathrm{ODsamples}-\mathrm{ODcontrol}}{\mathrm{ODnormal}-\mathrm{ODcontrol}}\times 100\%$$

### *In vitro* identification of antiphotoaging effects

#### UV irradiation model

As a source of UVB radiation, a UV lamp (peak, 311 nm; TL20W/01, Royal Dutch Philips Electronics Ltd., Amsterdam, Netherlands) was used. The HaCaT cells were seeded in a 96-well plate at a density of 1 × 10^5^ cells/well and then cultured in 200 μL of DMEM containing 10% FBS for 24 h. Fresh DMEM without FBS (200 μL) was added to each well. Then, the UV lamp was used to irradiate the samples at different times. Subsequently, the MTT assay was used to evaluate cell viability to select a suitable UV irradiation dose.

#### UVB irradiation recover assay

HaCaT cells were inoculated in a 96-wells plate at a density of 1 × 10^5^ cells/well for 24 h, modeled by a UV lamp (8.64 mJ/cm^2^), and treated with MITC, MITC/NPs, HACE/NPs, and HACE/MITC NPs (20 μg/mL) for 24 or 48 h. The MTT assay was used to evaluate cell viability.

#### ELISA assay

HaCaT cells were inoculated in a 96-wells plate for 24 h, modeled by a UV lamp (8.64 mJ/cm^2^), and treated with MITC, MITC/NPs, HACE/NPs, and HACE/MITC NPs (20 μg/mL) for 24 h. The GSH, MDA, SOD, and ROS released from cell culture supernatants were investigated using an ELISA kit (Beijing Solarbio Science & Technology Co., Ltd., Beijing, China) as per the manufacturer’s instructions. The color reaction was developed using a chromogenic agent, and the optical density was measured using a microplate reader.

#### Western blot analysis

Cells were modeled by the UV lamp (8.64 mJ/cm^2^) and treated by MITC, MITC/NPs, HACE/NPs, and HACE/MITC NPs (20 μg/mL) for 24 h when drugs placed in 1 day, 5 days and 10 days. The total cell protein was extracted using a radioimmunoprecipitation assay buffer (Biyuntian Biotechnology Co., Ltd., Shanghai, China). The BCA assay kit was used to assess protein concentrations. Cell lysates contained 20 ng of protein per lane and were separated using sodium dodecyl sulfate-polyacrylamide gel electrophoresis, transferred by electroblotting to polyvinylidene difluoride membranes (Millipore, Billerica, MA), and treated with MMP1, MMP3, and MMP9 for western blotting. Further, β-actin was used to normalize the total protein amount. A signal was detected using an ECL western blotting substrate and analyzed using ImageJ (Bethesda, MD).

### Statistical analysis

A *t*-test was used to analyze the statistical significance of the data. *p* Values <.05 and <.01 indicate significant and extremely significant differences, respectively. The results are shown as mean ± SD.

## Results and discussion

### Synthesis and characterization of HACE

**Figure 1.**
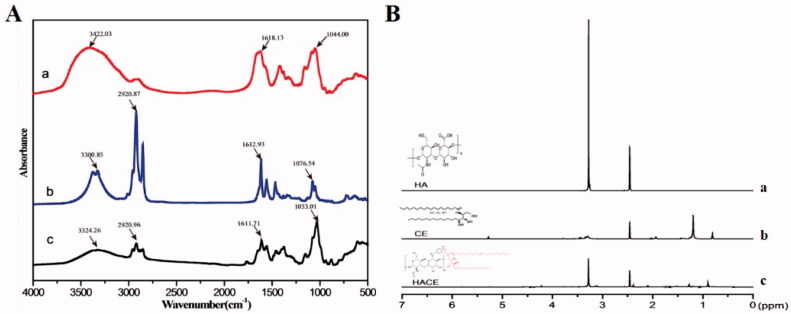
Ceramide modified by hyaluronic acid was characterized via Fourier transform infrared spectroscopy (FTIR) and nuclear magnetic resonance hydrogen spectroscopy (^1^H NMR); both proved the successful grafting of hyaluronic acid to ceramide. (A) FTIR and (B) ^1^H NMR.

The main component of the ECM, HA, is widely used in drug delivery (Cho et al., 2011) because it is biocompatible, biodegradable, nontoxic, non-immunogenic, and rich in carboxyl and hydroxyl functional groups. Further, CE, which is composed of sphingosine and fatty acids, is a component of cellular membranes and known to be involved in the regulation of differentiation, proliferation, programmed cell death, and apoptosis (Saddoughi et al., 2008). Figure 1(A) shows the obvious absorption peaks at 3324.26 cm^−1^ for HA, which correspond to the sugar hydroxyl group. The peak at 2920.96 cm^−1^ for CE belongs to the methylene group. The peak at 1611.71 cm^−1^ indicates the absorption of the secondary amide; compared with HA and CE, the secondary amide absorption of HACE shows a red shift. The FTIR results confirm that HACE is successfully synthesized. The peaks for the glucuronic acid group of HA (3.2 ppm) and the methyl group of CE (0.9 ppm) were confirmed in the ^1^H NMR spectra, which indicate the successful synthesis of HACE (Figure 1(B)).

### Preparation and characterization of flexible nanoliposomes

**Figure 2.**
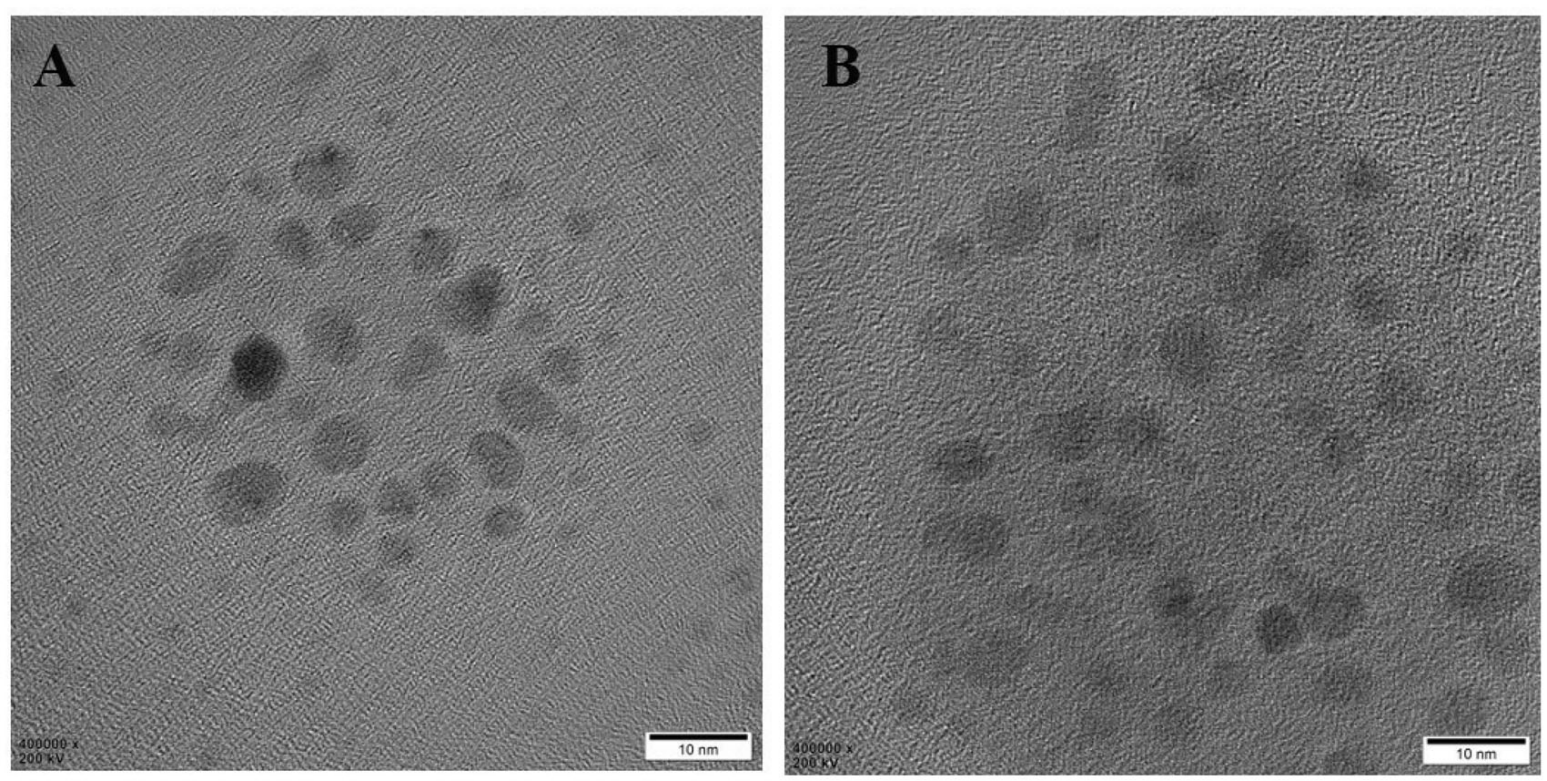
Transmission electron microscopy image of nanoparticles. (A) MITC NPs and (B) HACE/MITC NPs.

The HACE-modified MITC nanoliposomes were prepared for targeted MITC delivery to transdermal absorption. MITC, with its poor water solubility and oxidation susceptibility, was incorporated in the internal region of HACE/NPs (Tumer et al., 2015). As an amphiphilic HA derivative, HACE was introduced to the nanoliposomes, and MITC was loaded into a flexible nanoliposome formulation by the reported solvent evaporation method (Cho et al., 2012). The diameters of the blank, HACE, MITC-loaded (MITC NPs), and HACE MITC-loaded liposomes (HACE/MITC NPs) were 278, 198, 266, and 192 nm, respectively; the polydispersity index values of both MITC NPs and HACE/MITC NPs were approximately 0.3, which indicate a narrow size distribution (Table 1). In the TEM images, similar particle sizes and spherical morphologies of MITC NPs and HACE/MITC NPs were observed, and MITC NPs and HACE/MITC NPs were both round (Figure 2). The zeta potential values of both MITC NPs and HACE/MITC NPs were negative. The mean EE% values of MITC in NPs and HACE/NPs were 62.54% and 70.67%, respectively.

**Figure 3.**
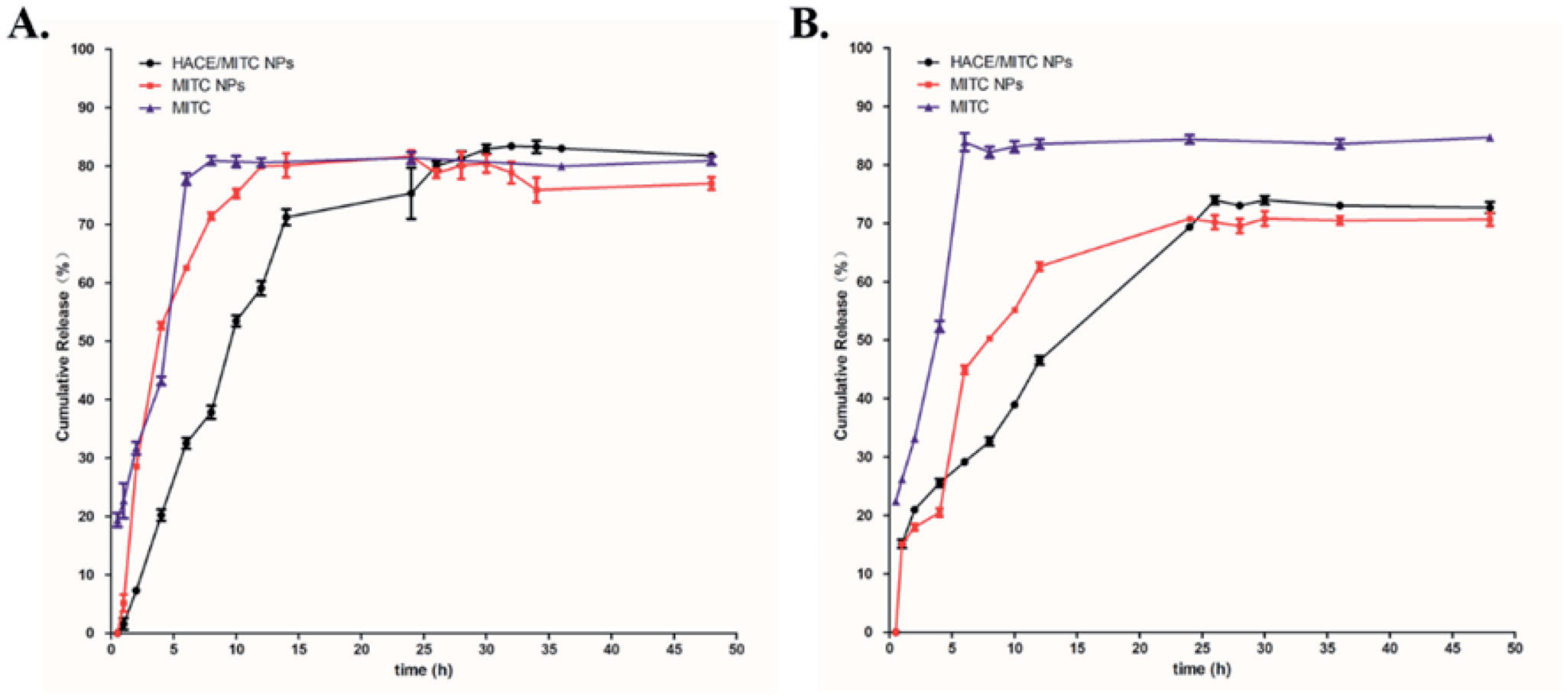
*In vitro* release rates of two types of nanoliposomes and pured MITC. Drugs were placed in (A) PBS (pH 7.4), (B) PBS (pH 5.5) at 25 °C; the release pattern was monitored for 48 h at 25 °C under shaking at 100 rpm.

The *in vitro* release profiles of MITC from MITC NPs, HACE/MITC NPs, and pured MITC were assessed at PBS (pH 7.4) and PBS (pH 5.5) respectively in room temperature (Figure 3). In the medium of pH 7.4, compared with the rapid release feature of pured MITC (about 81% within 8 h), at 14 h, the two types of nanoliposomes explosive release occurred, and the release rate slowed down. The cumulative release of MITC in MITC NPs was approximately 80.10% at 24 h, and the amount of release reached its maximum. However, the release rate of MITC in HACE/MITC NPs increased slowly after 24 h, and the cumulative release reached a maximum of 83.39% until 32 h. In the medium of pH 5.5, compared with the rapid release feature of pured MITC (about 83.9% within 6 h), MITC NPs appeared explosive release at 14 h, and the cumulative release of MITC in MITC NPs was the maximum release of approximately 70.81% at 24 h. HACE/MITC NPs explosive release occurred at 26 h, the cumulative release of MITC in HACE/MITC NPs was the maximum release of approximately 73.97% in 26 h. Therefore, the *in vitro* cumulative release rate indicates that the sustained release effect of HACE/MITC NPs was better than that of MITC NPs. Besides, in different medium, the drug release rate of NPs is greatly affected in different pH.

### Effects of nanoliposomes on the stability of MITC

The MITC should be stored at 4 °C and away from light; otherwise, it degrades easily because of the influence of inappropriate environmental factors such as temperature and light (Waterman et al., 2014). Therefore, the stability of MITC is a major concern to its application. The change in drug content was detected at different time points at room temperature (25 °C) to investigate the stability of MITC encapsulated by liposomes.

**Figure 4.**
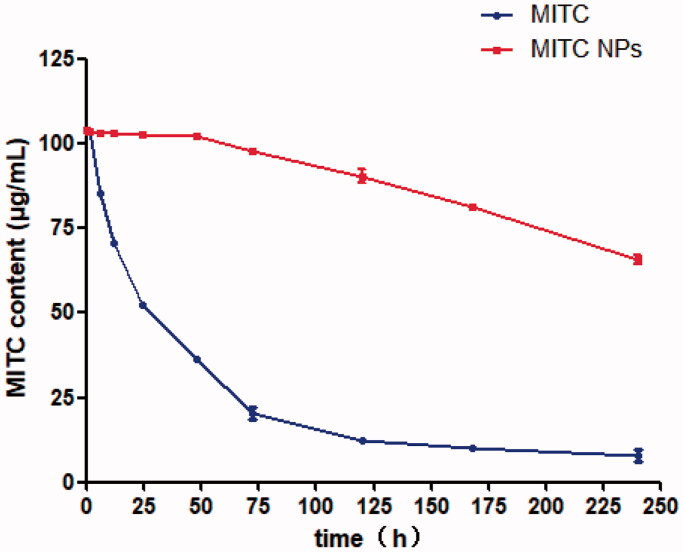
Stability of MITC liposomes and MITC solution at 25 °C. The MITC liposomes were tested by a low temperature ultracentrifugation method.

Figure 4 shows the change in the drug content of MITC and MITC NPs at room temperature (25 °C). From 0 to 72 h, the drug content of MITC decreased from 104 to 19 μg/mL. After 72 h, the MITC content steadily dropped to 7 μg/mL at 240 h. The unsuitable temperature has a significant influence on the degradation of MITC. However, when the MITC was loaded with liposomes, the drug stabilized at 102 μg/mL before 48 h, and after 72 h, the drug content dropped to 98 μg/mL. The drug MITC content in the liposome dropped to 65 μg/mL at 240 h. The MITC content of the MITC NPs was still ninefolds higher than that of the MITC drug at 240 h. These results indicate that liposomes can prevent drug degradation. Although the stability of MITC NPs is better than that of MITC, the retention period of MITC remains too short for storage. Further, the enhancement of drug stability via liposome composite drug delivery systems is a trend (Yuan et al., 2021), and it is expected to be the research direction for our team in the future.

### *In vitro* skin permeation study

The percutaneous application of isothiocyanates is difficult because of the instability and hydrophobicity of isothiocyanates (Nakamura et al., 2019). However, HA and CE have good transdermal performance (Lee et al., 2017), and the combination with MITC liposomes can theoretically enhance their transdermal absorption rate. Therefore, we used an *in vitro* transdermal test to detect the transdermal performance of HA and CE combined with MITC NPs and HACE MITC.

Table 2 summarizes the percentage of drug retention and penetration in the skin after 24 h. The accumulated drug of MITC NPs is larger than that of HACE/MITC NPs, which are believed to have better skin penetration performance than MITC NPs and to be easily able to penetrate the skin cuticle and enter the deep skin to deliver the drug. Meanwhile, the drug permeability and total drug delivery rate of the HACE/MITC NPs group were higher than those of MITC NPs; this can be attributed to the skin permeability and drug release ability of particles being enhanced by HACE, which improves transcutaneous drug delivery (How et al., 2020; Uche et al., 2021).

**Table 2.** Cumulative permeability and retention of MITC in the liposome after 24 h of *in vitro* diffusion experiments (*x*±SD, *n* = 3).

Sample	Accumulated drug (%)	Permeated drug (%)	Total drug delivered (%)
MITC NPs	15.77 ± 1.87	49.42 ± 1.42	65.19
HACE/MITC NPs	8.54 ± 1.2	71.40 ± 1.7	79.49

**Figure 5.**
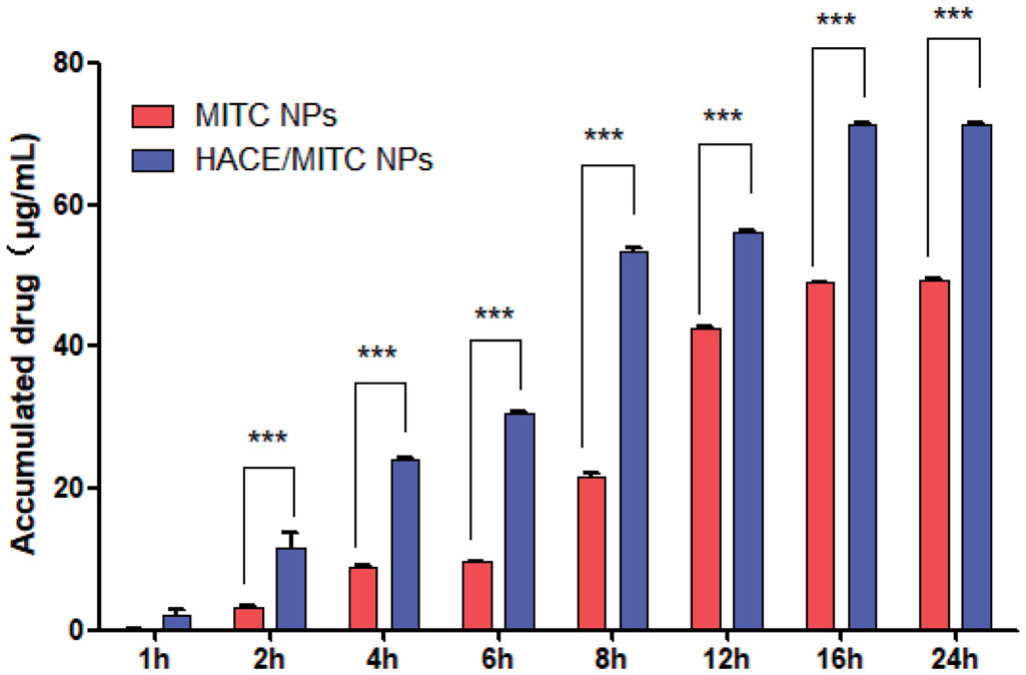
Cumulative penetration amount of MITC in liposomes varies with the penetration time (*x*±SD, *n* = 3). Comparison of MITC NPs and HACE MITC/NPs, ***p < .005.

Figure 5 shows the cumulative permeation of MITC NPs and HACE/MITC NPs at 1, 2, 4, 6, 8, 12, 16, and 24 h. At each time point, the cumulative permeations of HACE MITC NPs were higher than those of MITC NPs, which were significantly different at all time points except at 1 h (*p*<.005). Between 6 and 16 h, the permeability of MITC NPs was weaker than that of HACE/MITC NPs, and the permeability of HACE/MITC NPs was only 49.41 μg/cm^2^ after 24 h; the permeability of HACE/MITC NPs was stronger and the cumulative permeations reached 71.40 μg/cm^2^ after 24 h. The cumulative permeation of HACE/MITC NPs was 1.4-fold higher than that of the MITC NPs.

### Intracellular uptake of HACE/MITC NPs

**Figure 6.**
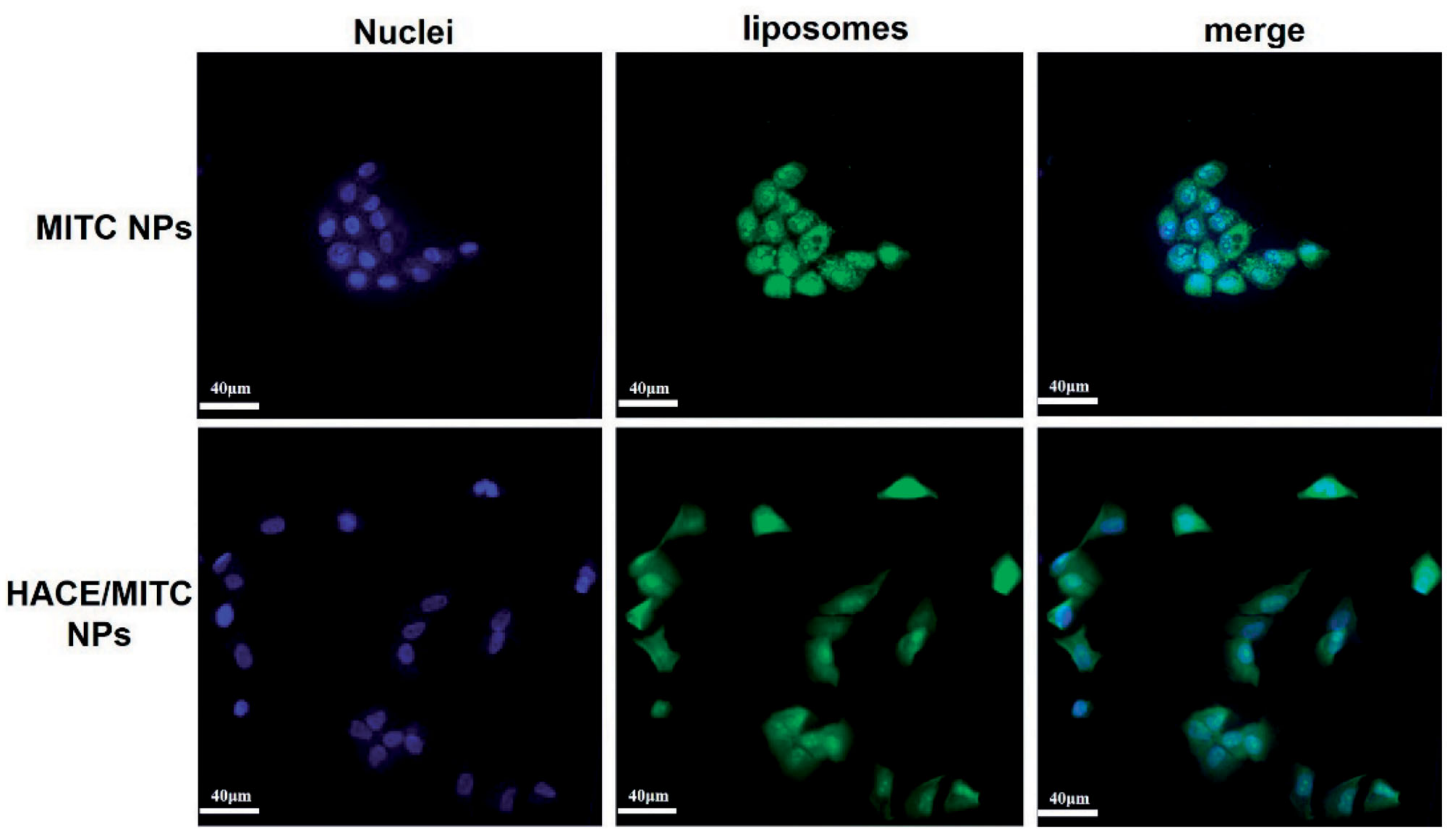
Intracellular uptake of two types of nanoparticles. Cell uptake of the two types of nanoparticles treated with FITC fluorescence liposomes for 4 h. After cell fixation, the cells were stained with DAPI, and observed and photographed in confocal microscopy.

The uptake of HACE/MITC NPs by HaCaT cells affects skin photoaging. The MITC NPs and HACE/MITC NPs carrying FITC were prepared and used to study the uptake of HACE/MITC NPs by HaCaT cells *in vitro* for confirming that HACE/MITC NPs prepared in this study can be taken up by HaCaT cells. Both MITC NPs and HACE/MITC NPs carrying FITC showed strong green fluorescence, which indicates that liposomes contributed to cell uptake. However, MITC NPs seriously affected cell morphology and did not appear in HACE/MITC NPs (Figure 6). The results showed that HACE/MITC NPs were favorable for uptake by HaCaT cells without changing cell morphology.

### Cytotoxicity and protective effect of HACE/MITC NPs against UVB-induced cell death

The main routes of drug penetration through the skin include cuticle hair follicles and sweat ducts, which are the latter two pathways often referred to as bypasses because the cutaneous appendage accounts for less than 1% of the entire skin surface area. Further, it is not the main absorption pathway in most cases (Karadzovska et al., 2013). As the main pathway, the stratum is a multilayer dense membrane structure that comprises keratinocytes and their lipid components in a narrow space. The stratum corneum cell membrane is not a lipid bilayer structure but a dense cross-linked protein network structure. The dense structure formed by multiple keratinocytes is the main obstacle to drug absorption through the skin (Ita, 2014). Therefore, it is believed that the main barrier to transdermal penetration is the stratum corneum. The strong lipophilicity of MITC, has an obvious role in the skin cuticle barrier, short skin attachment time, and low permeability.

**Figure 7.**
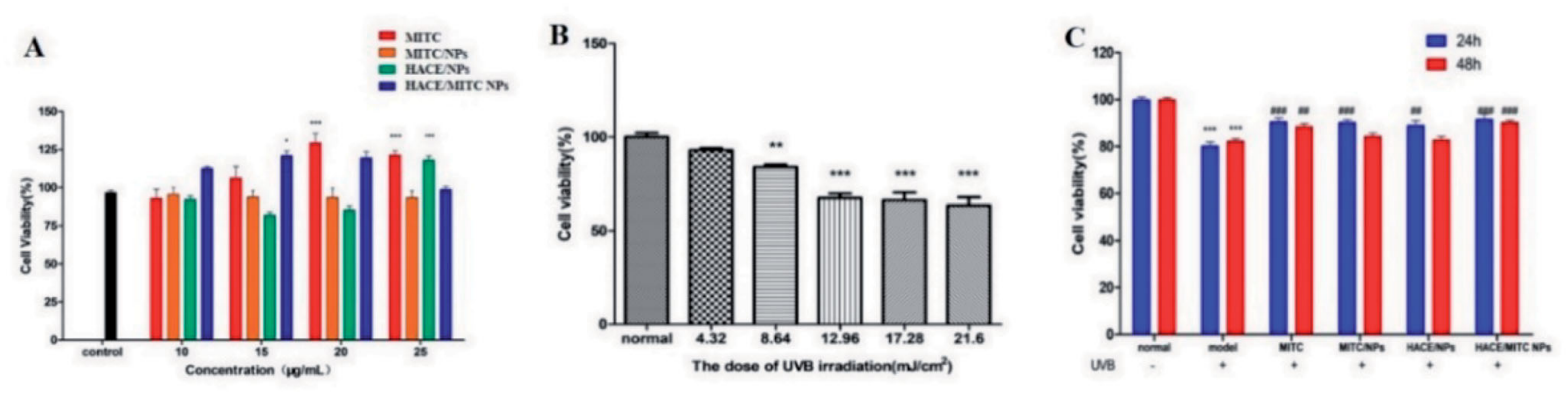
MTT assay to evaluate the effect of cell viability under different conditions. (A) Effects of different drugs on the viability of HaCaT cells at 20 μg/mL; (B) effects of different UVB radiation doses on the viability of HaCaT cells; (C) effect of different drug treatment time on the viability of HaCaT cells after UVB injury (*n* = 6, *compared with the normal group, ***p < .001; ^#^compared to the UVB group, ^#^*p* < .05, ^##^*p* < .01, ^###^*p* < .001).

We prepared HACE/MITC NPs to reduce the barrier effect of the skin cuticle on MITC, which improved the skin adhesion and release properties of MITC to improve its anti-photoaging efficacy. We first verified the toxicity and cytoprotective effects of MITC, MITC/NPs, HACE/NPs, and HACE/MITC NPs to evaluate the effect of MITC incorporation in nanoliposomes and its ability to counteract HaCaT cell damage promoted by UVB. The samples were not toxic to HaCaT cells at all concentrations evaluated (10–25 μg/mL), and the MITC-carrying nanoliposomes demonstrated the proliferation of HaCaT cells (Figure 7(A)). As shown in Figure 7(B), UVB irradiation significantly decreased cell viability, and cell viability was 83% when the dose of UVB irradiation was 8.64 mJ/cm^2^. Therefore, 8.64 mJ/cm^2^ was selected as the UVB irradiation modeling condition. As expected, only MITC showed a cytoprotective effect at 24 and 48 h. A significant increase in cell viability was observed for MITC NPs, HACE/NPs, and HACE/MITC NPs at 24 h; however, a significant increase was observed only in HACE/MITC NPs at 48 h.

### HACE/MITC NPs restored antioxidant enzyme activity reduced by UVB

**Figure 8.**
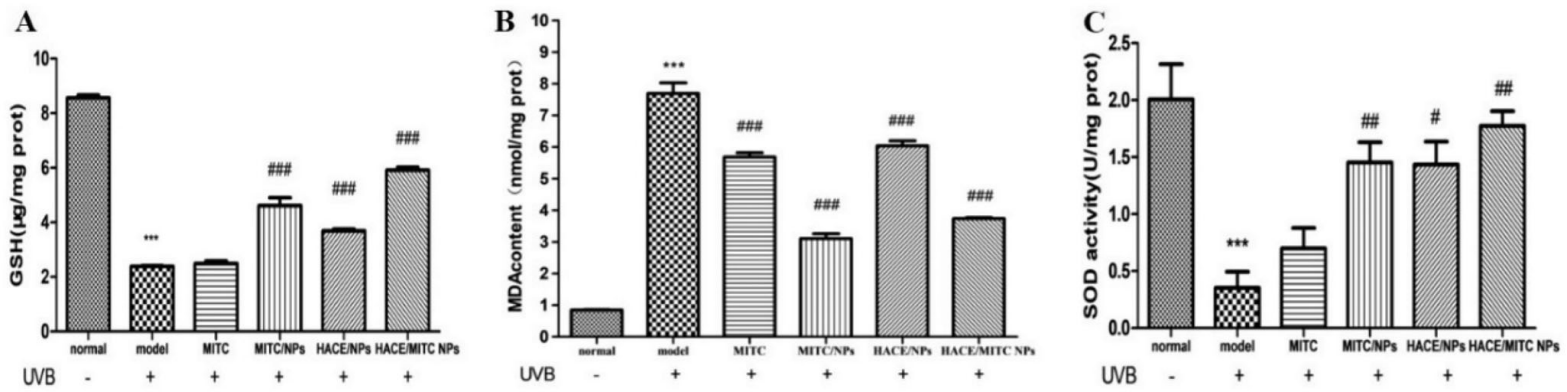
ELISA assay to evaluate the effects of antioxidant activity of the modified isothiocyanate liposome when drugs stored 10 days. (A) GSH content in different groups. (B) MDA content in different groups; (C) SOD activity in different groups (*n* = 4, *compared with the normal group, ***p < .001; ^#^compared to the UVB group, ^#^*p* < .05, ^##^*p* < .01, ^###^*p* < .001).

The activities of SOD, GSH-Px, and MDA were measured in the present study because previous studies indicated that antioxidative enzymes resist the skin from UV-induced photodamage by scavenging ROS (Herrling et al., 2006). It was observed that the activities of GSH-Px and SOD sharply decreased after continuous UV exposure compared to the normal control group (both *p* < .001), and the MDA was significantly increased (*p* < .001). However, when treated with MITC, MITC NPs, HACE/NPs, and HACE/MITC NPs, the decrease in GSH-Px and SOD and the increase in MDA induced by UV-irradiation were mitigated gradually, whereas HACE/MITC NPs had the best effect in that the activities of the antioxidative enzymes were closest to those of the normal group. Thus, HACE/MITC NPs had a certain ability to maintain the balance of oxidation and antioxidation in HaCaT cells. It is worth noting that the activities expression of antioxidant enzymes in drugs varies greatly with the storage time of drugs. When drugs were stored for one day (Fig. S1(A)) and five days (Fig. S2(A)), the GSH of each drug group showed strong activity. When drugs were stored to 10 days (Figure 8(A)), the MITC group basically had no GSH activity. But compared with the UVB group, MITC NPs, HACE/NPs, and HACE/MITC NPs groups still maintained strong GSH activity. In the assay of MDA, MDA elevation caused by UVB irradiation could be significantly reduced in MITC, MITC NPs, HACE/NPs, and HACE/MITC NPs after stored for one (Fig. S1(B)), five (Fig. S2(B)), and 10 (Figure 8(B)) days. Moreover, in the assay of SOD, the activity of the pured MITC was always low at one day (Fig. S1(C)), five days (Fig. S2(C)), and 10 days (Figure 8(C)), and it could not cure the decrease of SOD activity caused by UVB irradiation. However, the MITC NPs, HACE/NPs and HACE/MITC NPs groups had significant effects. This may be due to the SOD activity of lecithin in liposome preparation material (Ishihara et al., 2014). Therefore, when MITC was encapsulated, its can effectively restore antioxidant enzyme activities reduced by UVB.

### Effects of HACE/MITC NPs on reactive oxygen species generation and mitochondrial membrane potential

**Figure 9.**
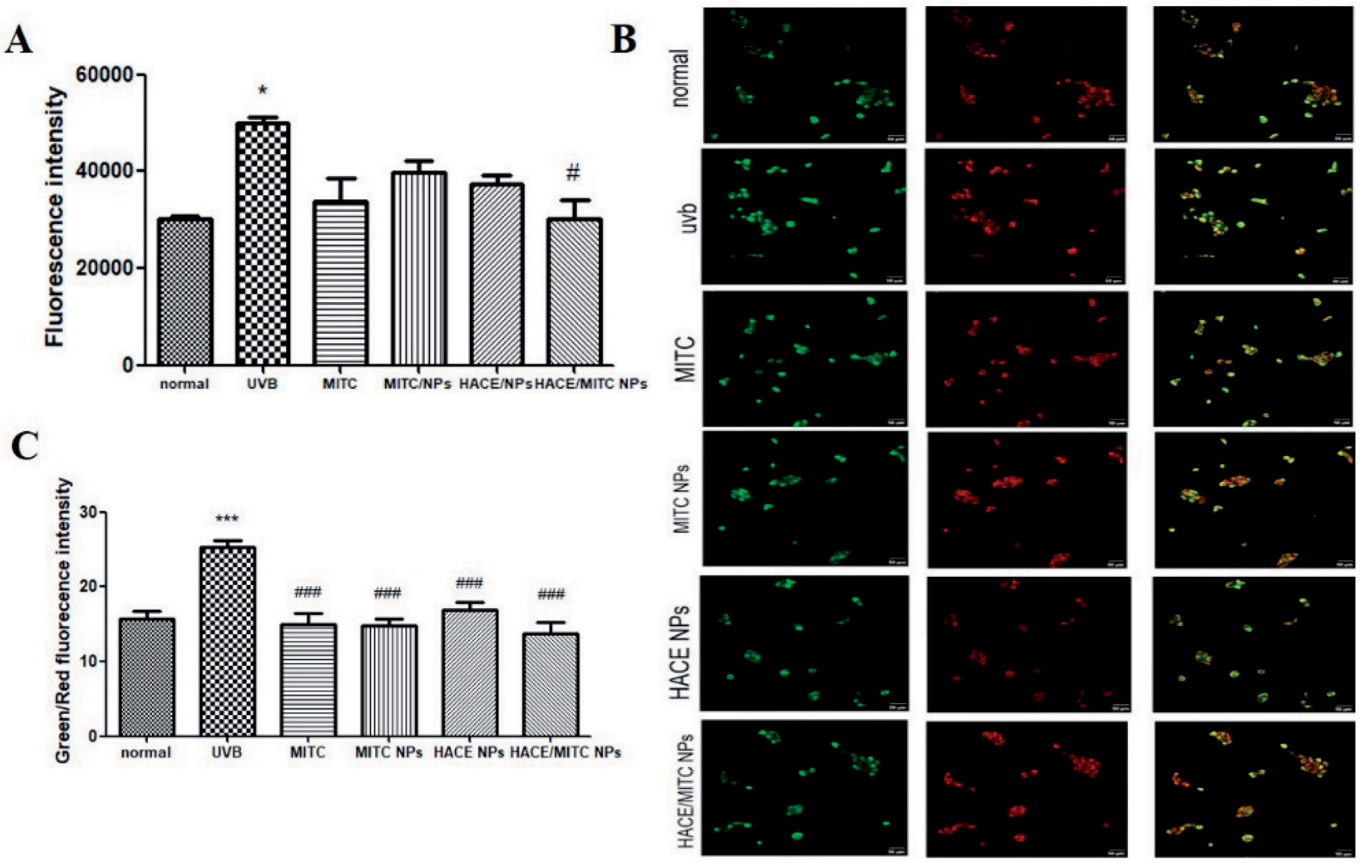
(A) ROS levels of MITC, MITC NPs, HACE/NPs, and HACE/MITC NPs on ROS generation in UVB-irradiated HaCaT cells; (B) effects of MITC, MITC NPs, HACE/NPs, and HACE/MITC NPs on the mitochondrial membrane potential in UVB-irradiated HaCaT cells; (C) green/red fluorescence intensities of each group. Results are expressed as the mean ± SD of three independent experiments; ***p < .001, *p < .05, vs. the UVB group, ^###^*p* < .001, ^#^*p* < .05 vs. the normal group.

As indicated in Figure 9(A), there is a significant increase in ROS levels in cells exposed to UVB radiation compared with ROS levels of the normal group. However, the cells treated with MITC, MITC NPs, HACE/NPs, and HACE/MITC NPs decreased the production of ROS, and the HACE/MITC NPs group showed significant inhibition of ROS activation compared with those for the UVB group. The results demonstrated that HACE/MITC NPs can scavenge UVB-induced ROS and protect the skin from damage.

Mitochondrial membrane potential (MMP) plays an important role in apoptosis and ROS generation. The MMP was measured to further detect the effect of MITC, MITC NPs, HACE/NPs, and HACE/MITC NPs on UVB-induced HaCaT cells. The results are shown in Figure 9(B). An increase in red fluorescence and a decrease in green fluorescence are observed after treatment with MITC, MITC NPs, HACE/NPs, and HACE/MITC NPs; these results demonstrate that the antioxidant role of HACE/MITC NPs in UVB-exposed HaCaT cells is related to the rescue of the mitochondrial function.

### HACE/MITC NPs reduced the high expression of MMPs caused by UVB

**Figure 10.**
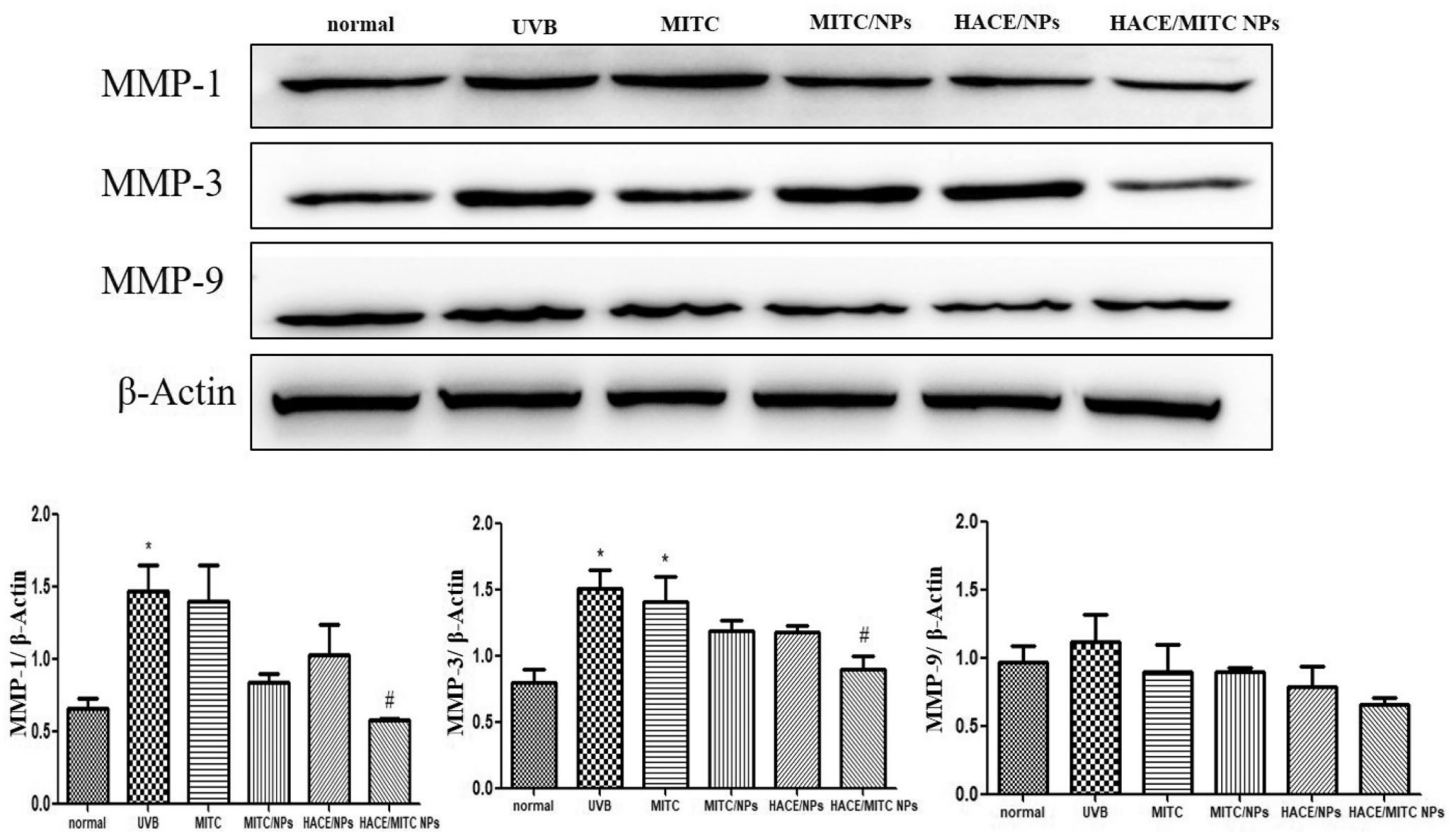
Western blotting was used to evaluate the UVB-induced reduction of photoaging of the modified isothiocyanate liposomes when drugs stored 10 days. (A) Protein expression of MMP-1; (B) protein expression of MMP-3; (C) protein expression of MMP-9. Data are expressed as mean ± SEM from three independent experiments (*n* = 3, *compared with the normal group, *p < .05; ^#^compared to the UVB group, ^#^*p* < .05).

Proteolytic enzymes such as MMPs and elastases are produced by epidermal keratinocytes and fibroblasts in the mediation of ECM remodeling (Philips et al., 2011). The MMPs initiate the photoaging of the skin by acting as collagenases (Mu et al., 2021). Collagen and elastin are the major structural proteins in the ECM. The basal levels of the enzymes increase under various conditions such as aging; however, they increase considerably more under environmental pollutants and UV radiation. The UV radiation induces high expressions of MMP-1, MMP-3, and MMP-9. MMP-1 is the most important enzyme for degrading the components of the ECM and breaking the normal structure of collagen fibers and elastic fibers; MMP-3 is a stromelysin; and MMP-9 degrades denatured collagens (Quan et al., 2009). The changes of MMP1, MMP3, and MMP9 were not obvious when drugs were stored for one day (Fig. S3), five days (Fig. S4), and 10 days (Figure 10). MMP-1 is the most important enzyme for degrading the components of the ECM and breaking the normal structure of collagen and elastic fibers; MMP-3 is a stromelysin; and MMP-9 degrades denatured collagens. The expressions of MMP-1, MMP-3, and MMP-9 are significantly higher than those of the normal group (*p* < .05). Further, when treated with HACE/MITC NPs, the expressions were significantly decreased compared with those for the UVB group (*p* < .05).

## Conclusions

In this study, HA-CE was synthesized for the modification of nanoliposomes and flexible nanoliposome-coated MITC were prepared. The EE% of HACE/MITC NPs was 70.67%, and HACE/MITC NPs released MITC slowly, with a cumulative release rate of 83.39% in PBS (pH 7.4); when in PBS (pH 5.5) the cumulative release rate of 73.97%. The flexible nanoliposomes successfully improved the skin and cell membrane permeation of MITC and the effect of anti-photoaging. The *in vitro* photoaging experiment demonstrated that HACE/MITC NPs can markedly improve the activities of antioxidant enzymes, scavenge UVB-induced ROS, and reduce MMP-1, MMP-3, and MMP-9 expressions caused by radiation-induced photoaging. This suggests that HACE/MITC NPs may be useful as a candidate for photoaging therapy.

## Supplementary Material

Supplemental Material

## Data Availability

Not applicable.
