# Supplementary material for: Anti-photoaging effects of flexible nanoliposomes encapsulated Moringa oleifera Lam. isothiocyanate in UVB-induced cell damage in HaCaT cells
Source: Drug Deliv. 2022 Mar 11;29(1):871–81. doi: 10.1080/10717544.2022.2039802 (PMC8920399; doi:10.1080/10717544.2022.2039802)
Supplement: Supplemental Material [file IDRD_A_2039802_SM6515.docx]

**Supplemental Information**

**Anti-photoaging effects of flexible nanoliposomes encapsulated *Moringa oleifera Lam.* isothiocyanate in UVB-induced cell damage in HaCaT cells**

**Yijin Wang^1^, Qianqian Ouyang^1,4,5^*, Xuefei Chang^1^，Min Yang^1^, Junpeng He^1^, Yang Tian^1,2^* and Jun Sheng^3*^**

1. College of Food Science and Technology, Yunnan Agricultural University, Kunming, China

2. Engineering Research Center of Development and Utilization of Food and Drug Homologous Resources, Ministry of Education, Yunnan Agricultural University, Kunming, China

3. Yunnan Province Engineering Research Center of Functional Food of Homologous of Drug and Food ,Yunnan Agricultural University, Kunming, China

4. Marine Biomedical Research Institution, Guangdong Medical University, Zhanjiang, 524023, PR China

5. The Marine Biomedical Research Institute of Guangdong Zhanjiang, Zhanjiang 524023, China

**Corresponding author:**

*E-mail*s:shengj@ynau.edu.cn(Sheng Jun); tianyang1208@163.com(Tian Yang); [oyqq617@gdmu.edu.cn](mailto:oyqq617@gdmu.edu.cn)(Ouyang Qianqian)

**Table S1 the formulation composition**

| Composition | Lecithin/mg | Cholesterol/mg | sodium cholate/mg | HACE/mg | MITC/mmol/L |
| --- | --- | --- | --- | --- | --- |
| black liposome | 460 | 46 | 44 | - | - |
| HACE liposome | 460 | 46 | 44 | 30 | - |
| MITC-loaded liposome | 460 | 46 | 44 | - | 0.05 |
| HACE MITC-loaded liposome | 460 | 46 | 44 | 30 | 0.05 |


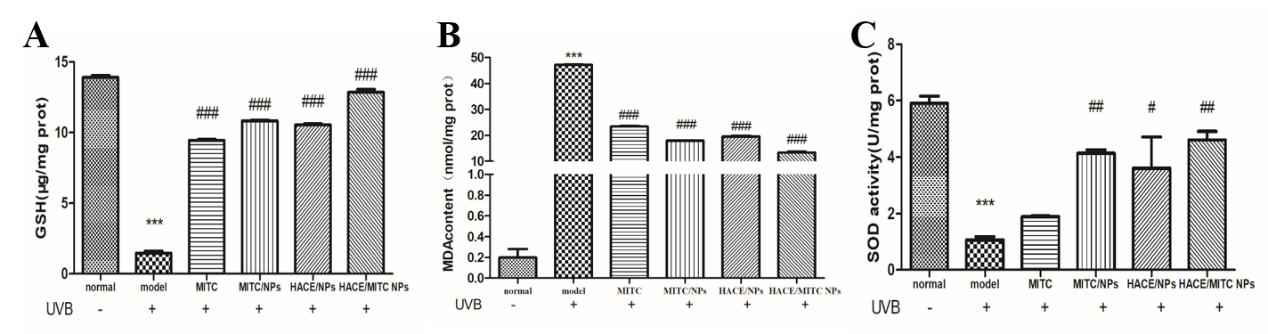


**Fig. S1** ELISA assay to evaluate the effects of antioxidant activity of the modified isothiocyanate liposome when drugs stored 1 day (25 ℃, in dark) . (A) GSH content in different groups. (B) MDA content in different groups; (C) SOD activity in different groups. (n = 4, * is compared with the normal group, ****p* < 0.001; # is compared to the UVB group, #*p* < 0.05, ##*p* < 0.01, ###*p* < 0.001)


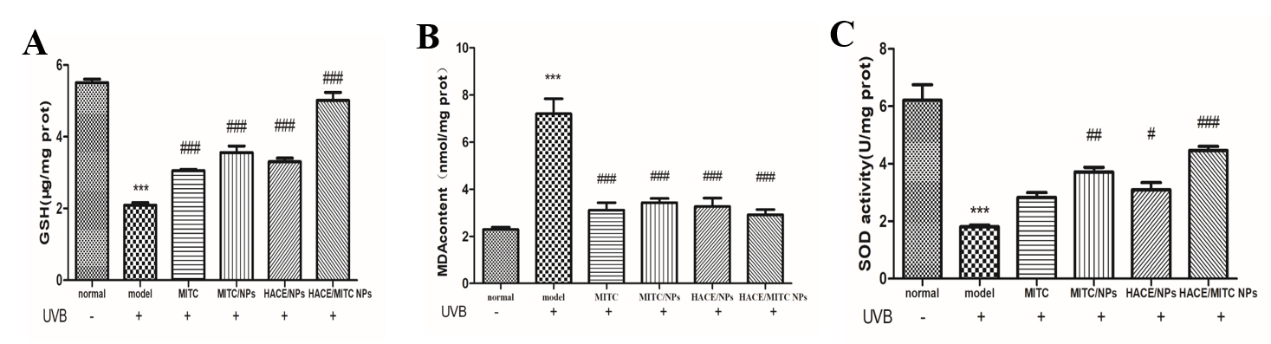


**Fig. S2** ELISA assay to evaluate the effects of antioxidant activity of the modified isothiocyanate liposome when drugs stored 5 days (25 ℃, in dark) . (A) GSH content in different groups. (B) MDA content in different groups; (C) SOD activity in different groups. (n = 4, * is compared with the normal group, ****p* < 0.001; # is compared to the UVB group, #*p* < 0.05, ##*p* < 0.01, ###*p* < 0.001)


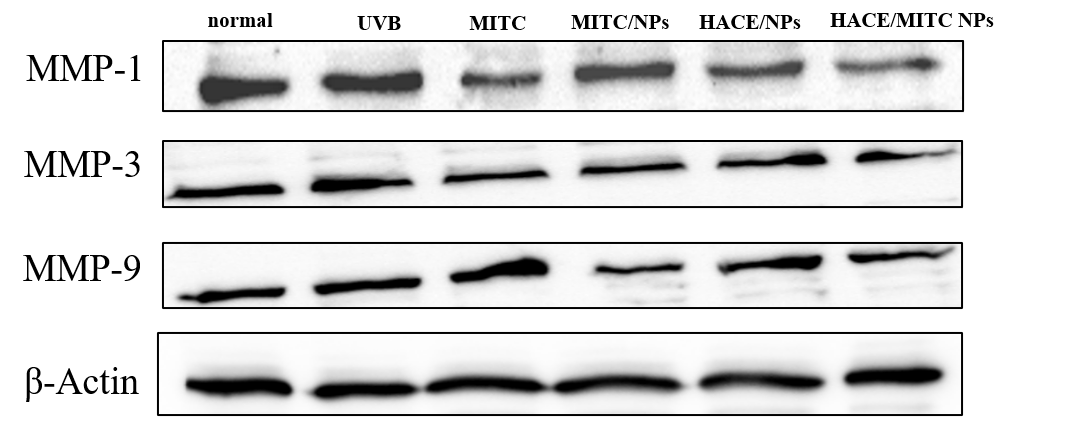


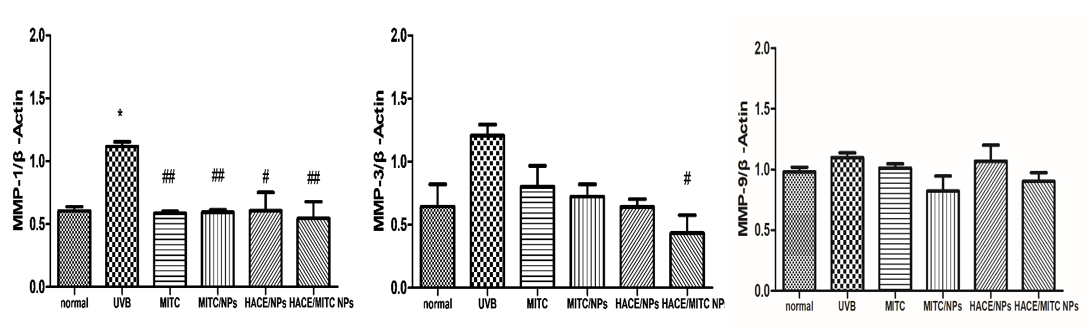


**Fig. S3** Western blotting was used to evaluate the UVB-induced reduction of photoaging of the modified isothiocyanate liposomes when drugs stored 1 day. (A) Protein expression of MMP-1; (B) Protein expression of MMP-3; (C) Protein expression of MMP-9. Data are expressed as mean ± SEM from three independent experiments (n = 3, * is compared with the normal group, **p* < 0.05; # is compared to the UVB group, #*p* < 0.05).


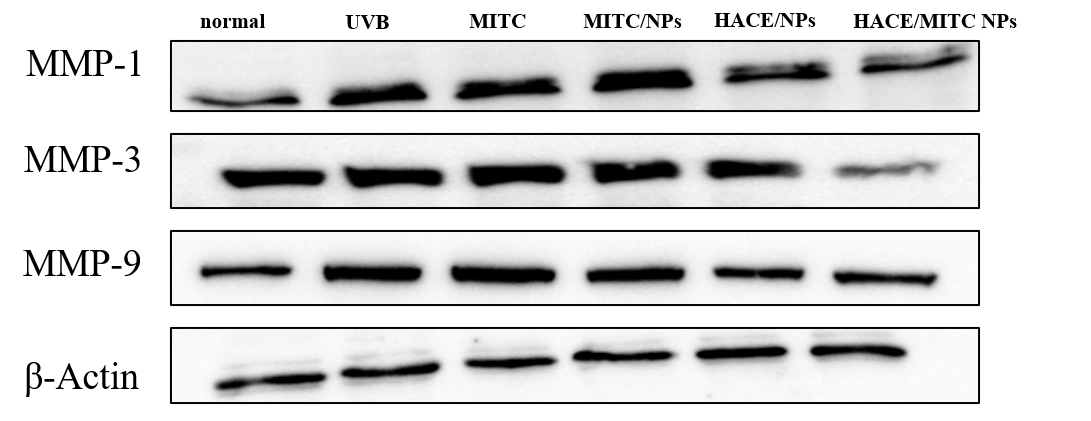


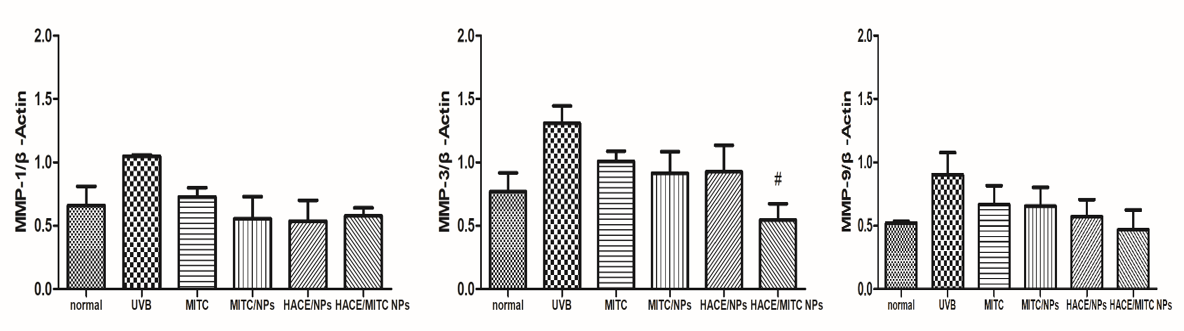


**Fig. S4** Western blotting was used to evaluate the UVB-induced reduction of photoaging of the modified isothiocyanate liposomes when drugs stored 5 days. (A) Protein expression of MMP-1; (B) Protein expression of MMP-3; (C) Protein expression of MMP-9. Data are expressed as mean ± SEM from three independent experiments (n = 3, * is compared with the normal group, **p* < 0.05; # is compared to the UVB group, #*p* < 0.05).
